# Establishing a dominant early larval sex-selection strain in the Asian malaria vector* Anopheles stephensi*

**DOI:** 10.1186/s40249-024-01256-7

**Published:** 2024-11-11

**Authors:** Shih-Che Weng, Fangying Chen, Ming Li, Sammy Lee, Connor Gerry, Dylan Can Turksoy, Omar S. Akbari

**Affiliations:** School of Biological Sciences, Department of Cell and Developmental Biology, University of California, San Diego, La Jolla, CA 92093 USA

**Keywords:** Genetic sex sorting, Malaria, Doublesex, Sex-specific alternative splicing, *Anopheles stephensi*, Genetic biocontrol

## Abstract

**Background:**

Genetic biocontrol interventions targeting mosquito-borne diseases require the release of male mosquitoes exclusively, as only females consume blood and transmit pathogens. Releasing only males eliminates the risk of increasing mosquito bites and spreading pathogens while enabling effective population control. The aim of this study is to develop robust sex-sorting methods for early larval stages in mosquitoes, enabling scalable male-only releases for genetic biocontrol interventions.

**Methods:**

To address the challenge of sex-sorting in the Asian malaria vector *Anopheles stephensi*, we engineer Sexing Element Produced by Alternative RNA-splicing of a Transgenic Observable Reporter (SEPARATOR). This dominant fluorescent-based method, previously proven effective in *Aedes aegypti*, exploits sex-specific alternative splicing of a reporter to ensure exclusive male-specific expression early in development. The sex-specific alternative RNA splicing of the *doublesex* gene was selected as a target for engineering SEPARATOR due to its evolutionary conservation in insects. To expand SEPARATOR’s applicability for genetic sexing, we assessed the cross-species sex-specific RNA splicing activity of the *An. gambiae doublesex* (*AngDsx*) splicing module in *An. stephensi*. Male-specific enhanced green fluorescent protein (EGFP) expression was verified throughout the mosquito life cycle using a fluorescent stereomicroscope.

**Results:**

Our results confirm that SEPARATOR regulates male-specific EGFP expression in *An. stephensi* and enables reliable positive male selection from the first instar larval stages. Molecular analysis demonstrates that male-specific EGFP expression is dependent on *doublesex* sex-specific splicing events. Additionally, the splicing module from *An. gambiae* operates effectively in *An. stephensi*, demonstrating evolutionary conservation in sex-specific splicing events between these species.

**Conclusions:**

SEPARATOR’s independence from sex-chromosome linkage provides resistance to breakage that could be mediated by meiotic recombination and chromosomal rearrangements, making it highly suitable for mass male releases. By enabling precise male selection from the first instar larval stages, SEPARATOR represents a significant advancement that will aid in the genetic biocontrol for *Anopheles* mosquitoes.

**Graphical Abstract:**

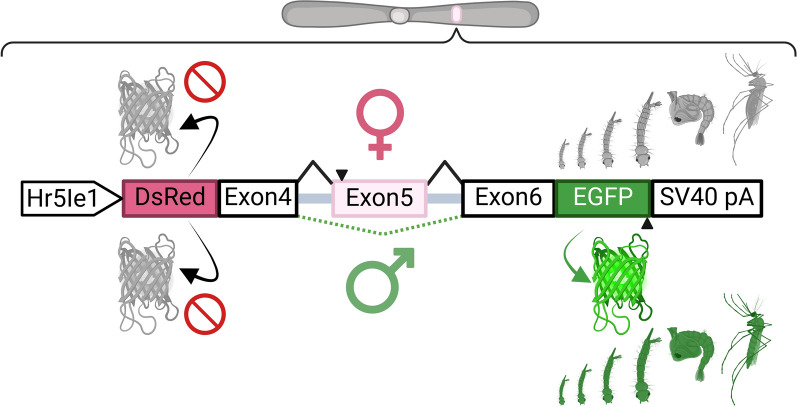

**Supplementary Information:**

The online version contains supplementary material available at 10.1186/s40249-024-01256-7.

## Background

Mosquitoes are the deadliest creatures on Earth, responsible for over a million deaths annually [1]. *Anopheles* mosquito species stand out as they transmit malaria, one of the most widespread and lethal diseases, claiming hundreds of thousands of lives annually [2, 3]. While malaria control has traditionally targeted rural Africa, there has been an increasing concern about its transmission in rapidly urbanizing cities in Africa [3–5]. This urban transmission is increasing with the invasion of *Anopheles stephensi*, a mosquito species notorious for its ability to transmit both of the significant *Plasmodium* malaria parasites, *P. falciparum* and *P. vivax* [6–8]. Originally native to South Asia and the Arabian Peninsula, *An. stephensi* has been detected in seven African countries to date [6–8]. During a malaria outbreak in Dire Dawa, Ethiopia, between April–July 2022, *P. falciparum* infections in malaria patients were strongly associated with the presence of *An. stephensi* in the household vicinity, which also tested positive for *Plasmodium* sporozoites [6–8]. Furthermore, climate change is expanding the habitable ranges of mosquitoes and their pathogens, resulting in an increasing number of people at risk of contracting mosquito-borne diseases [9–12]. Innovative technologies targeting these mosquitoes are essential for addressing this global challenge [13–16].

Genetic biocontrol strategies for *Anopheles* mosquito populations have primarily focused on male sterilization and female suppression, particularly in the context of population control [13, 14, 16, 17]. Radiation-based sterile insect technique (SIT) involves releasing sterile males into the wild, where they mate with females but do not produce viable offspring [18, 19]. Repeated releases of sterile males can gradually reduce the overall mosquito population over time [18, 19]. However, SIT has had limited success for *Anopheles* species due to difficulties associated with sex sorting and fitness costs resulting from mass rearing and irradiation. To overcome these limitations, the precision-guided sterile insect technique (pgSIT) has been implemented in *An. gambiae*, utilizing genetic modifications to enable simultaneous male selection and sterilization [20]. By using clustered regularly interspaced short palindromic repeats (CRISPR)/CRISPR-associated protein 9 (Cas9) targeting female essential genes and male fertility genes, pgSIT achieves female elimination and male sterilization avoiding the need to sex sort and irradiate the released individuals [20]. Other genetic modification methods for biocontrol have been developed with a focus on suppressing the female mosquito in the population since females are the primary vectors for pathogen transmission through blood meals. Methods include creating a male-biased population using gene drive technology to reduce females in the population [21–25]. For example, homing endonucleases like intron-encoded endonuclease from *Physarum polycephalum* (I-PpoI nuclease) and the CRISPR and Cas9 system are used to target the X chromosome or essential sex determination genes such as *doublesex* (*dsx*), effectively reducing female offspring. Other methods include the use of a drug-repressible system that will eliminate females in the absence of the drug allowing for the release of fertile males [26], and a binary CRISPR approach eliminates females by targeting the female-essential gene *femaleless* [27]. By achieving female elimination and male sterilization, these strategies aim to effectively disrupt mosquito reproduction cycles, indicating that a promising direction for biocontrol is to suppress females and select males carrying biocontrol modules, thus spreading these traits in the population. While the above methods show promise in controlling mosquito populations and reducing disease transmission, the implementation of a scalable and efficient sex-separating method is required to significantly enhance the application of these genetic strategies [28, 29].

The pursuit of effective mosquito sex separation methods in *Anopheles* species has been met with progress and challenges. In *Anopheles* species, the primary method for sex separation is by observing the external morphology of the mosquitoes. However, the  differences between male and female *Anopheles* mosquitoes are subtle and challenging to discern, especially without specialized equipment or training. To address this significant limitation, scientists have genetically associated selectable markers with the Y chromosomes of *Anopheles* mosquitoes [30–32]. In addition to Y-linked markers, sex-specific promoters have also been utilized. For example, Catteruccia et al utilized a testis specific promoter to drive male-specific EGFP expression during late larval development, allowing for the identification of male mosquitoes by the third instar larval stage [32]. Alternatively, a method utilizing biased EGFP expression to differentiate *Anopheles* larvae has been developed in *An. gambiae*, improving the efficacy of the sex-sorting marker [33, 34]. However, these methods encountered challenges as the expression patterns lacked significant divergence for effective separation, particularly during the early instar larvae stages. To better implement the genetic biocontrol techniques, a reliable, scalable, and efficient sex-separating method that enables efficient early larval sex sorting is essential.

In this study, we focus on advancing the genetic biocontrol approach tailored for mosquito *An. stephensi*. To develop a robust sex-sorting method for *An. stephensi*, we utilize the Sexing Element Produced by Alternative RNA-splicing of A Transgenic Observable Reporter (SEPARATOR), which has been previously applied to *Aedes aegypti, Ceratitis capitata, Drosophila melanogaster, Drosophila suzukii* [35, 56]. By leveraging the conserved sex-specific splicing patterns observed in *Anopheles* mosquitoes, our method demonstrates targeted male selection. Moreover, given the versatility of SEPARATOR across various species within the genus, our method enhances its utility for exploring biocontrol strategies tailored for *An. stephensi* and related species.

## Methods

### Molecular cloning and transgenesis

Plasmids were constructed using the Gibson enzymatic assembly technique. DNA fragments were amplified from existing plasmids and genomic DNA of the *An. gambiae* G3 strain using Q5 Hotstart High-Fidelity 2 × Master Mix (New England Biolabs, Ipswich, USA). To construct the AngDsx splicing module, we initially amplified the fragment containing endogenous exons and introns from the genomic DNA of *An. gambiae* using PCR. All primers (Integrated DNA Technologies, San Diego, USA) are listed in Table S2. We then linearized the *Ae. aegypti* SEPARATOR plasmid, Vector 1174D, which includes DsRed and EGFP and is available from Addgene (ID: 200012). This linearized Vector 1174D plasmid was combined with the amplified exons and introns from *An. gambiae* genomic DNA in a Gibson enzymatic assembly to produce Vector 1174 K. The resulting plasmids were introduced into chemically competent Zymo JM109 *Escherichia coli* (Zymo Research, Tustin, USA), amplified, and purified using the Zymo Research Zyppy plasmid miniprep kit (Zymo Research, Tustin, USA). They were then sequenced using Sanger sequencing. The chosen plasmids underwent maxi-preparation with the ZymoPURE II Plasmid Maxiprep kit (Zymo Research, Tustin, USA) and were sequenced extensively using Oxford Nanopore Sequencing (Plasmidsaurus, San Francisco, USA). The SEPARATOR plasmid (Vector 1174 K), derived from the *AngDsx* splicing module, and its annotated DNA sequence map is available at Addgene (ID: 221017).

Transgenic lines were created by injecting preblastoderm stage embryos with a mixture of the piggyBac plasmid and a transposase helper plasmid. The injected G0 embryos were left to melanize for two more days before being floated in trays. Surviving pupae were sorted by sex. Male and female pupae were placed in separate cages, with a 5:1 ratio of wild-type males to females. A blood meal was provided after mating, and eggs were collected, aged, and hatched. Larvae with positive fluorescent markers were identified using a fluorescent stereomicroscope. To identify unique insertion events, transformants with fluorescent markers were bred with wild-type mosquitoes, creating distinct lines. To enhance the homozygous population, the SEPARATOR lines underwent approximately ten generations of sibling matings, selecting individuals with the most vibrant marker expression in each generation.

### Mosquito rearing and maintenance

*An. stephensi* mosquitoes of a strain (UCISS2018), which was previously used to generate the reference genome, was used in this study [36]. The mosquitoes were reared in incubators set at 28 °C with 20–40% humidity and a 12-h light/dark cycle, housed in cages (BugDorm, Taichung, China) measuring 24.5 × 24.5 × 24.5 cm. Adult mosquitoes had access to 10% (m/V) aqueous sucrose solution ad libitum. Females were provided a blood meal by feeding on anesthetized mice for approximately 15 min, and oviposition substrates were introduced about three days after the blood meal. Eggs were allowed to melanize for an additional two days before being floated in trays. Larvae were reared in plastic containers (Sterilite, Townsend, USA) containing approximately three liters of deionized water and were fed fish food (TetraMin, Blacksburg, USA). For genetic crosses, female virginity was ensured. Pupae were sexed and separated, relying on sex-specific morphological differences in the genital lobe shape (located at the end of the pupal abdominal segments, just below the paddles) before releasing them into cages. These general rearing procedures were consistently followed unless otherwise specified.

To increase the number of homozygotes in the SEPARATOR transgenic line, both high-intensity EGFP pupae and female EGFP-negative pupae were transferred to a cage and allowed to mate after eclosion. Female mosquitoes were provided a blood meal, and five adult females were individually transferred to egg tubes for colonization and egg collection. Eggs from each colony were hatched and reared. Colonies with a higher proportion of female EGFP negatives and male EGFP positives were selected for colonization in the subsequent generation.

### Fluorescent sorting, sexing and imaging

Mosquitoes were examined and imaged using the Leica M165FC fluorescent stereomicroscope equipped with the Leica DMC2900 camera (Leica Microsystems, Wetzlar, Germany). We used a Leica DM4B upright microscope equipped with a VIEW4K camera for higher-resolution images. To distinguish between male and female pupae in mosquitoes, we observed the sex-specific morphological differences in the genital lobe shape located at the end of the pupal abdominal segments just below the paddles.

### Y chromosome-linked gene detection

To examine the sex of SEPARATOR at the larval stage, we isolated genomic DNA from individual mosquitoes in both EGFP-positive and EGFP-negative groups using the Blood & Cell Culture DNA Midi Kit (Qiagen, Venlo, Netherlands). Primers specific to Y chromosome-linked genes were employed in PCR to determine male mosquitoes among both EGFP-positive and EGFP-negative larvae [37].

### Determination of genome integration sites

To identify the integration sites of the transgene, inverse PCR and Sanger DNA sequencing methods were employed according to previous studies with modifications [38]. Genomic DNA was isolated from 5 EGFP-positive and 5 EGFP-negative mosquitoes at the larval stage of SEPARATOR using the Blood & Cell Culture DNA Midi Kit (Qiagen, Venlo, Netherlands), adhering to the provided instructions. Digestion of roughly 1 μg of SEPARATOR genomic DNA was carried out using *Hae*III or *Taq*I-v2 enzymes. The digested samples were then purified and ligated overnight at 16 °C with T4 DNA ligase. This ligation mixture was subsequently utilized as a template for PCR amplification. Specific primers were synthesized to amplify the genomic regions flanking the transgene's left and right arms. The PCR-amplified fragments were then purified through gel extraction and sequenced. The sequencing data were analyzed using BLAST to align with the *An. stephensi* genome [36], allowing for the precise determination of the transgene insertion sites by comparing the alignments from both arms. Finally, we designed a confirmed primer pair that anneals upstream and downstream of the insertion sites in the genome, enabling the amplification of the full length of the transgene element within the mosquito genome.

### Detection of sex-specific RNA splicing

Total RNA was extracted from 10 EGFP-positive and 10 EGFP-negative mosquitoes at the pupal stage utilizing the miRNeasy Tissue/Cells Advanced Mini Kit (Qiagen, Venlo, Netherlands), in accordance with the prescribed procedures of the manufacturer. The genomic DNA was removed with the aid of the genomic DNA eliminator column that comes with the kit. The reverse transcription was performed using both random primers and oligo dT primers to generate a comprehensive cDNA pool. To identify the sex-specific alternative splicing of the *AngDsx* splicing module, RT-PCR was utilized. Primers were specifically crafted to anneal to the terminal regions of the *Hr5IE1* promoter and the initial segment of the EGFP gene (Table S2). The PCR amplifications were then subjected to Sanger sequencing for detailed analysis.

## Results

### Engineering SEPARATOR for precise male mosquito sorting

**Fig. 1:**
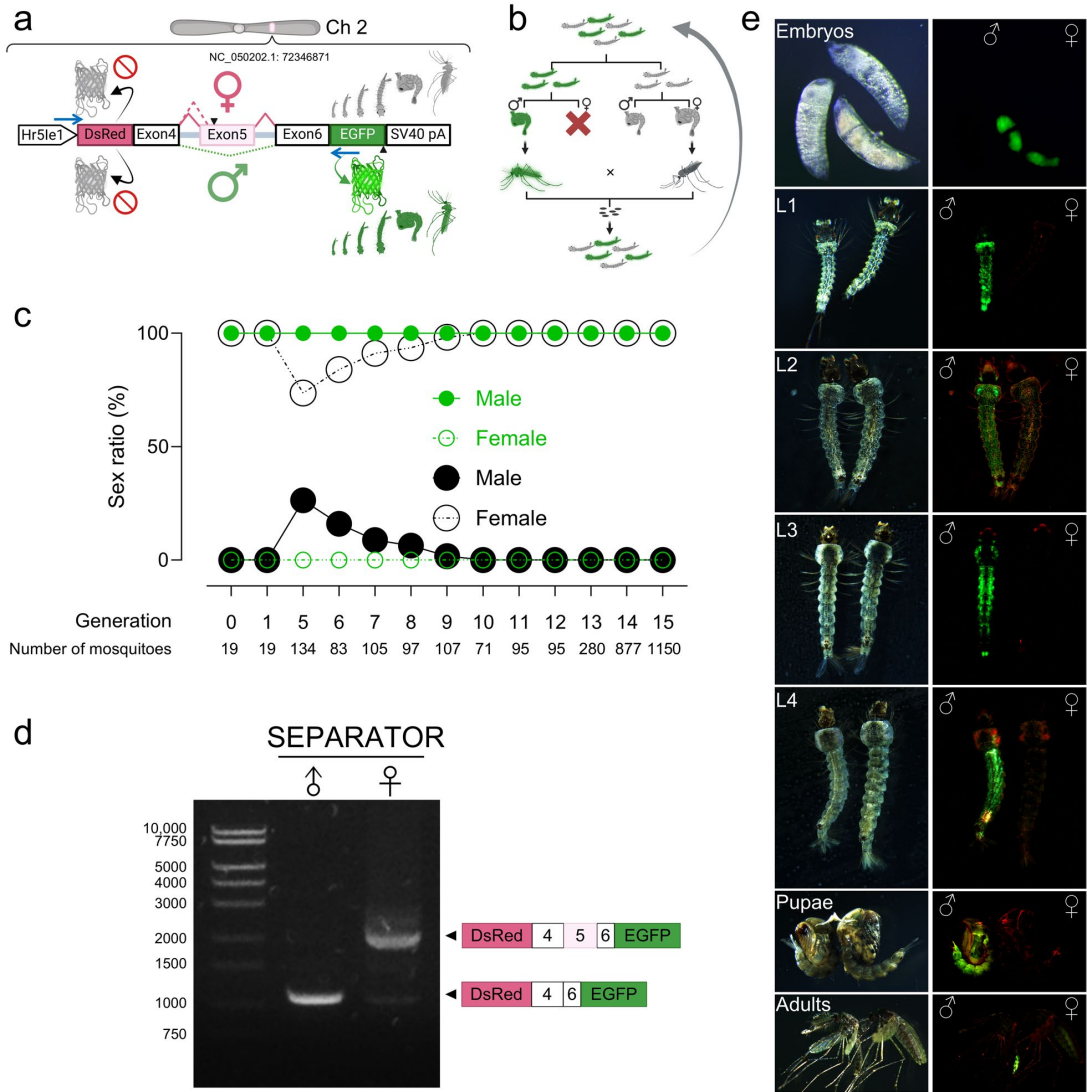
SEPARATOR in *Anopheles stephensi*. **a** The sex-specific splicing module of *An. gambiae dsx* (*AngDsx*) was used to construct SEPARATOR in *An. stephensi.* The expression of SEPARATOR was regulated by the *Hr5Ie1* promoter, which is a constitutive promoter derived from a baculovirus. In males, the splicing process generated a specific product that was compatible with the EGFP coding sequence. To ensure sex-specific expression, stop codons were strategically kept in exon 5, effectively preventing the in-frame expression of EGFP in female organisms. The SV40 pA sequence enabled proper termination and processing of the SEPARATOR transcript. The blue arrows indicate the relative positions of the primer target sites used for RT-PCR. The construct is not to scale. **b** EGFP-positive mosquitoes are crossed with EGFP-negative female mosquitoes to maintain and increase the percentage of homozygous offspring. **c** Sex ratios were determined within GFP-positive and GFP-negative larvae across generations (GFP-positives are: in green symbol; GFP-negatives are in black). Sex was determined by examining the morphological differences in genital lobe shape at the pupal stage using a microscope, which are specific to each sex (male: closed circle; female: open circle). **d** Sex-specific RNA splicing of SEPARATOR. Ten EGFP-positive and ten EGFP-negative mosquitoes at the pupal stage were used. Subsequently, total RNA was extracted from each group. To investigate the splicing patterns, RT-PCR was conducted using specific primers that targeted the 3′ end of the *Hr5Ie1* promoter sequence and the 5' end of the EGFP coding sequence. The PCR products underwent agarose gel electrophoresis, followed by gel purification and sequencing to confirm the splicing patterns. The resulting splicing patterns are shown in the right panel. **e** The developmental stages of SEPARATOR mosquitoes, including embryo, larva, pupa, and adult, were imaged using a fluorescent stereomicroscope (Leica M165FC). The images are presented in two panels: the upper panel displays bright-field images, while the lower panel shows the GFP/mCH channel images. *SEPARATOR* Sexing Element Produced by Alternative RNA-splicing of a Transgenic Observable Reporter, *AngDsx Anopheles gambiae doublesex*, *Hr5Ie1* AcMNPV homologous region 5 enhancer and immediate early gene 1 promoter, *EGFP* Enhanced green fluorescent protein, *SV40 pA* Simian vacuolating virus 40 poly(A) signal, *RT-PCR* Reverse transcription polymerase chain reaction

In this study, we investigated the potential of applying SEPARATOR for genetic sexing in *An. stephensi*, leveraging a sex-specific splicing module derived from the *An. gambiae dsx gene* (*AngDsx*)*.* This approach was based on the hypothesis that the evolutionary conservation of the *dsx* gene would enable its functionality across species (Fig. S1). The transcriptional regulation of these constructs was mediated by the constitutive *Hr5IE1* AcMNPV baculovirus promoter, which has proven effective across a wide range of species [39–46]. The reading frame was initialized by incorporating a start codon along with a Kozak sequence, aligned in-frame with the DsRed coding sequence within the transgenic mosquito. Translation of DsRed halts due to the stop codon in the female-specific exon, regulated by female-specific RNA splicing in females. We hypothesize that in male mosquitoes, the stop codon within the female-specific exon will be spliced out, allowing the DsRed coding sequence to align in-frame with the EGFP coding sequence. This design activates male-specific EGFP expression, facilitated by male-specific RNA splicing (Fig. 1a). The initial objective was to ensure that all mosquitoes expressing EGFP would be male, while DsRed would serve as a dominant marker for transgenic mosquitoes, expressed in both sexes (Fig. 1a). Interestingly, following microinjection, three EGFP-expressing larvae were observed, all identified as male during the pupal stage in G0, but no DsRed-expressing larvae were observed in G0. Subsequently, all pupae from G0 were sexed, and resulting adults were crossed with wild-type mosquitoes to establish stable transgenic lines based on fluorescence markers in G1. From G1 onwards, consistent results were observed, with 100% of the whole body EGFP-expressing larvae being male, and no DsRed-expressing larvae were detected. Over the course of 15 generations, a total of 1556 EGFP-positive larvae were manually screened, and their resulting sex at the pupal and adult stages was confirmed. Remarkably, 100% of the EGFP-positive larvae were male (Fig. 1c, Table S1). Our findings demonstrate that the male-specific splicing module from *AngDsx* can splice and result in male-specific expression of EGFP in *An. stephensi*, confirming the evolutionary conservation of sex-specific RNA splicing elements between *An. gambiae* and *An. stephensi*.

### Male-specific EGFP expression throughout the entire life cycle of SEPARATOR mosquitoes

In generation 9, 100% of the EGFP-positive larvae were confirmed as genetic males by verifying the presence of the Y chromosome at the larval stage, with their sex further validated at both the pupal and adult stages (Fig. S2). However, 1 out of 6 EGFP-negative larvae was identified as a Y chromosome-containing wild-type male, while the remaining 5 were females (Fig. S2). This indicates that the population had not yet reached complete homozygosity by this generation. We therefore performed pairwise crosses for another three generations and confirmed the establishment of a healthy homozygous line by generation 12. Taken together, these results demonstrated that SEPARATOR effectively separated male mosquitoes, even within a heterogeneous population and a homozygous strain could be established in which all EGFP-positive larvae were confirmed to be transgenic males (Fig. 1c and Fig. S2).

To further assess the utility and robustness of SEPARATOR throughout the mosquito life cycle, we investigated the timing of EGFP expression. Our observations revealed robust EGFP signals from the late embryo stage to adulthood in mosquitoes (Fig. 1e). In total, 3138 larvae were manually screened, and their sex was confirmed at both the pupal and adult stages. These results demonstrated that the SEPARATOR mosquitoes are sufficiently able to sort out male mosquitoes from the early stages of the mosquito life cycle.

### Male-specific gene expression regulated by sex-specific alternative RNA splicing

We validated the sex-specific splicing pattern of the SEPARATOR system, derived from the *AngDsx* splicing module, in *An. stephensi*. Reverse transcription polymerase chain reaction (RT-PCR) and sequencing confirmed the anticipated sex-specific splicing in transgenic *An. stephensi* (Fig. 1d, Fig. S4, and Fig. S7). Sequencing results showed that both male and female spliced RNA transcripts contained DsRed coding sequences, in-frame with the first start codon of the splicing module, indicating that sex-specific splicing does not hinder DsRed expression at the transcript level. However, DsRed signals were not detected in the transgenic larvae (Fig. 1c and e).

In male mosquitoes, EGFP expression was confirmed to result from male-specific RNA splicing rather than from sex chromosome linkage. Inverse PCR revealed that the SEPARATOR construct had integrated into autosome, specifically chromosome 2, intersecting with the ASTEI20_038714 gene, identified as the 60S acidic ribosomal protein P0 (Fig. S3 and Fig. S6). These results demonstrate that male-specific EGFP expression in *An. stephensi* is driven by male-specific splicing of the transgene.

## Discussion

To be compatible with vector control strategies aimed at combating *Anopheles* mosquito-borne diseases, we utilized the early-stage sex sorter approach SEPARATOR for selecting transgenic males. This system derives alternative RNA splicing of the sex determination gene *dsx* in *An. gambiae* and selects males by recognizing positive EGFP expression for *An. stephensi*.

We successfully implemented the sex-specific *dsx* module from *An. gambiae* in *An. stephensi*, and confirmed that 100% of the EGFP-positive *An. stephensi* larvae with the SEPARATOR were male. This indicates precise and effective positive male selection. In contrast to our study, previous studies have utilized negative male selection methods, demonstrating genetic sexing strains using X chromosome-linked reporters and female-specific splicing-regulated reporter expression in *Anopheles* mosquitoes [33, 34]. For male-only releases, the positive male selection system is more robust than negative selection sexing systems. Compared to negative selection that requires the removal of 100% females to separate males from the mosquito population (such as sorting males by using female-specific *dsx* mRNA to remove females), we positively select male mosquitoes by male-specific *dsx* mRNA. This system enables the efficient identification and retention of male mosquitoes, simplifying the sorting process and reducing the necessity for complete female elimination. Consequently, it provides a more effective and less labor-intensive method for producing male-only populations. The retained females can then be reintroduced into the rearing colony for mass production purposes. Additionally, the positive selection method used by SEPARATOR avoids issues related to spontaneous DNA mutations that can hinder the accuracy of negative selection.

In addition to positive male selection, utilizing the SEPARATOR offers several advantages. First, the male-specific EGFP expression is not linked to the Y chromosome, which circumvents chromosomal recombination events typically observed during large-scale insect rearing and prevents disruption of any modules linked to the sex chromosomes [29, 47, 48]. Second, the earliest stage at which selection occurs is the late embryo stage. Compared to a previous study using testis-specific promoter-driven reporters in *An. stephensi*, which enabled the identification of male mosquitoes in the earliest at the third instar larvae [32], our study advances *An. stephensi* sex sorting to the first instar stage. This improvement is due to the fact that the *dsx* gene is integral to the broader sex determination pathway and is expressed in both males and females across various body tissues [49–52]. This widespread expression allows the module to function in multiple tissues and, when combined with ubiquitous promoters, enables expression in diverse tissues throughout developmental stages. As the SEPARATOR is not limited to specific tissues, it enhances the efficiency of fluorescence sorting at early developmental stages of mosquitoes. Being able to separate sex in such an early developmental stage contributes to major savings in food, space, time, and labor during mass rearing [28]. Third, as the SEPARATOR overcomes the limitations of tissue-specific promoters, which can result in fluorescence being localized to specific body parts, such as previously reported the sixth abdominal segment [32]. Ultimately, the successful application of the *dsx* module across species demonstrates its potential for transferability among different species. This success can be attributed to the evolutionary conservation of the *dsx* gene sequence and function.

During the course of our study, our results notably demonstrate that the female-specific exon 5 in SEPARATOR is shorter than the corresponding exon in *An. gambiae* by sequencing the female-specific mRNA splicing fragment [53]. This finding suggests that the female-specific RNA splicing of SEPARATOR in *An. stephensi* favors the splicing acceptor site within exon 5 of *An. gambiae* rather than the expected splicing acceptor site located in the intron region [53]. Bioinformatic analysis detected two female exon-containing splicing products by comparing RNA sequencing data against whole-genome sequencing data in *An. stephensi* [54, 55]. Our RT-PCR result with wild-type *An. stephensi* corroborates the bioinformatics analysis (Fig. S5), demonstrating the presence of two female exon-containing transcripts resulting from alternative 3ʹ splice-site selection on the female-specific exon (exon 5). One product contains the full-length exon 5, while the other contains a truncated version of exon 5, resulting in different C-terminal ends of the female-specific DSX protein. Interestingly, in *Ae. aegypti*, more than one female-specific transcript is produced by the female exons (exon 5a and exon 5b) selected through exon skipping [49]. This difference suggests a divergence in the evolution of sex-determination mechanisms within Diptera. However, the regulatory mechanisms and functional implications of the two splicing products have yet to be elucidated. Further investigation is warranted to reveal the alternative splicing mechanism of the *dsx* gene and enhance our understanding of sex determination in *Anopheles* mosquitoes.

Unexpected results were observed with the SEPARATOR construct, which was designed to facilitate DsRed expression in both male and female *An. stephensi*. However, no DsRed-positive mosquitoes were detected. Despite the *dsx* splicing module in SEPARATOR generating the expected sex-specific spliced transcripts and preserving the DsRed coding sequence in both male- and female-specific transcripts, no DsRed signals were observed in either sex. This might result from improper folding or functionality of the DsRed protein due to the fusion with the DSX peptide altering its structure or interfering with its normal folding process. As a result, while our system could precisely identify male mosquitoes at the early developmental stage, it could not reliably distinguish transgenic females from non-transgenic individuals in the heterozygous SEPARATOR mosquitoes due to the non-detectable DsRed signal. Further studies will continue to utilize SEPARATOR for precise identification of both males and females in *Anopheles* mosquito populations.

Despite challenges with female selection, the SEPARATOR leverages its unique features to ensure precise and efficient male-only selection. Consequently, SEPARATOR can significantly enhance mosquito control by enabling the selection of male-specific traits in both homozygous and heterozygous SEPARATOR mosquitoes. This is particularly useful for strategies such as releasing only sterile males, which can be applied in both conventional SIT and pgSIT. Since binary CRISPR approaches have been extensively studied in mosquitoes, such as pgSIT and Inherited Female Elimination by Genetically Encoded Nucleases to Interrupt Alleles (Ifegenia), SEPARATOR can be used to select males for either or both the Cas9 line and sgRNA lines at the larval stage. To further automate the SEPARATOR on a large scale, integrating it with the Complex Parametric Analyzer and Sorter (COPAS, Union Biometrica) presents a promising application. The COPAS instrument, known for its ability of sexing by using dominant reporter fluorescence, has been successfully used in our previous *Ae. aegypti* SEPARATOR work [35]. Therefore, by fully automating the sorting process, the SEPARATOR will enhance speed, facilitating the scalability of genetic technologies.

## Conclusions

The SEPARATOR system represents a significant advancement in genetic biocontrol for *Anopheles* mosquitoes. It effectively selects males through positive *dsx* mRNA-based identification, ensuring precise male selection. SEPARATOR represents a significant biocontrol advancement, ensuring accurate male selection and advancing genetic biocontrol efforts against mosquito-borne diseases.

## Supplementary Information

Supplementary Material 1: Figure S1. Comparing the intron structure of *doublesex* in *Anopheles gambiae* and *An. Stephensi. *Figure S2. EGFP-positive mosquitoes are Y chromosome-containing mosquitoes. Figure S3. The full length of SEPARATOR is inserted into the *Anopheles stephensi* genome. Figure S4. The sex-specific *dsx* transcripts align with sex-specific RNA splicing patterns in SEPARATOR mosquitoes. Figure S5. Two female-specific *dsx* transcripts were observed in *Anopheles stephensi*. Figure S6. Sequencing results related to Figure S3. Figure S7. Sequencing results related to Figure S4.

Supplementary Material 2: Table S1. The sex sorting of SEPARATOR mosquitoes. Table S2. Sequences of primers used in this study

## Data Availability

Complete sequence maps and plasmids are deposited at Addgene.org (#221017). All data used to generate figures are provided in the Supplementary Materials/Tables. Generated transgenic lines are available upon request to O.S.A.

## Abbreviations

SEPARATOR: Sexing Element Produced by Alternative RNA-splicing of a Transgenic Observable Reporter
*Dsx*: *Doublesex*
SIT: Sterile insect technique
pgSIT: Precision-guided sterile insect technique
CRISPR: Clustered regularly interspaced short palindromic repeats
Cas9: CRISPR associated protein 9
I-PpoI nuclease: Intron-encoded endonuclease from Physarum polycephalum
EGFP: Enhanced green fluorescent protein
DsRed: *Discosoma* Red fluorescent protein
Yg: Y chromosome-linked gene
AcMNPV: Autographa californica nuclear polyhedrosis virus
*Hr5IE1*: AcMNPV homologous region 5 enhancer and immediate early gene 1 promoter
SV40 pA: Simian vacuolating virus 40 poly(A) signal
RT-PCR: Reverse transcription polymerase chain reaction
COPAS: Complex Parametric Analyzer and Sorter
