# Supplementary material for: Establishing a dominant early larval sex-selection strain in the Asian malaria vector Anopheles stephensi
Source: Infect Dis Poverty. 2024 Nov 11;13:83. doi: 10.1186/s40249-024-01256-7 (PMC11552218; doi:10.1186/s40249-024-01256-7)
Supplement: Supplementary file 1 — Supplementary Material 1: Figure S1. Comparing the intron structure of doublesex in Anopheles gambiae and An. Stephensi. Figure S2. EGFP-positive mosquitoes are Y chromosome-containing mosquitoes. Figure S3. The full length of SEPARATOR is inserted into the Anopheles stephensi genome. Figure S4. The sex-specific dsx transcripts align with sex-specific RNA splicing patterns in SEPARATOR mosquitoes. Figure S5. Two female-specific dsx transcripts were observed in Anopheles stephensi. Figure S6. Sequencing results related to Figure S3. Figure S7. Sequencing results related to Figure S4. [file 40249_2024_1256_MOESM1_ESM.pdf]

Supplementary Figures

Figure S1. Comparing the intron structure of doublesex in *An. gambiae* and *An. stephensi*

Exon4

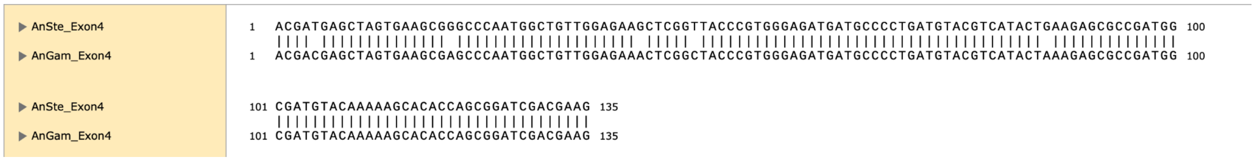

Exon5

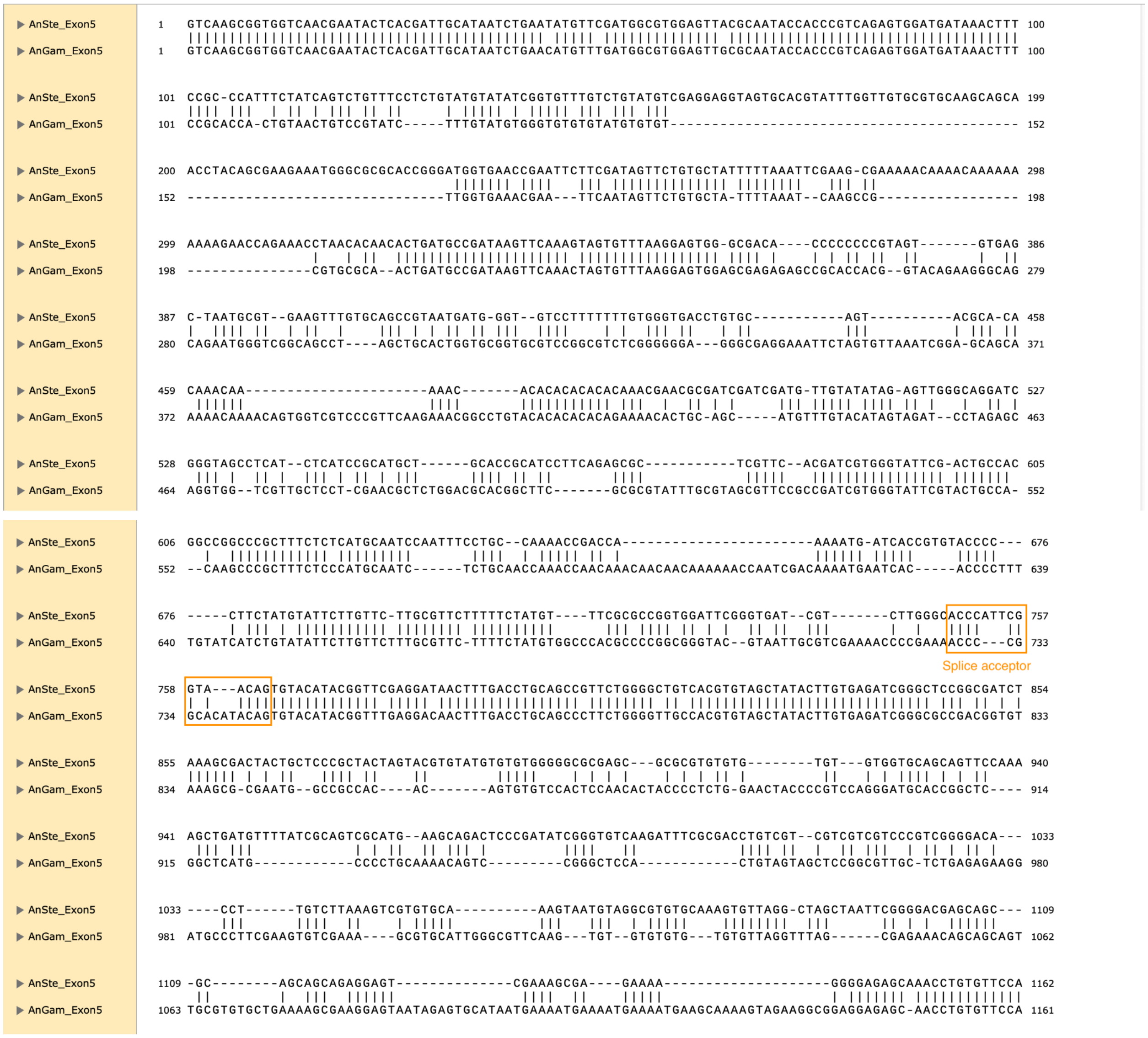

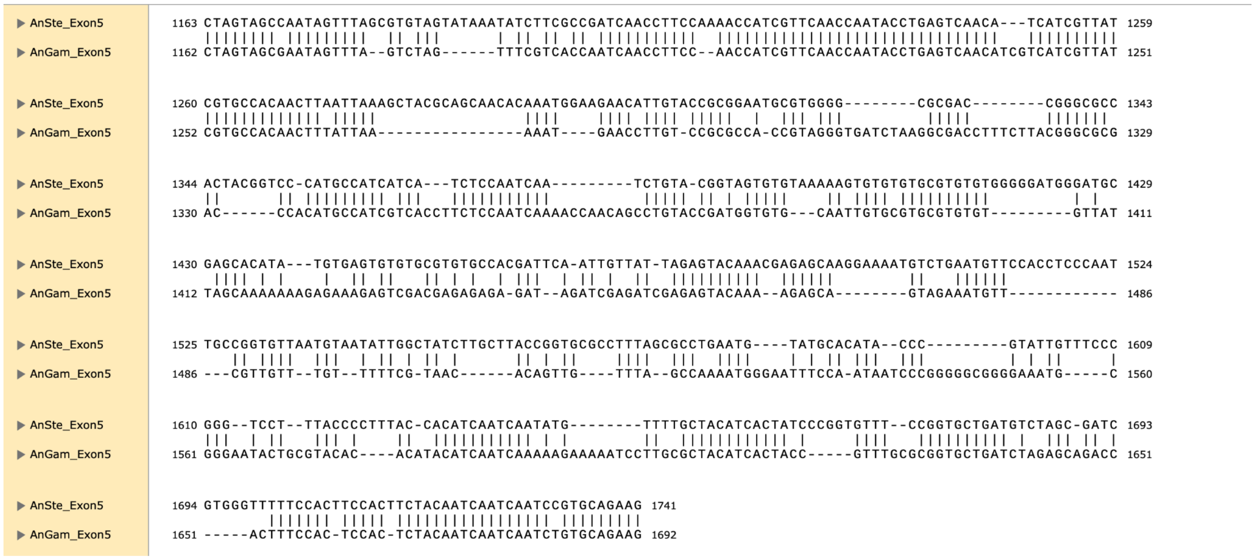

Exon6

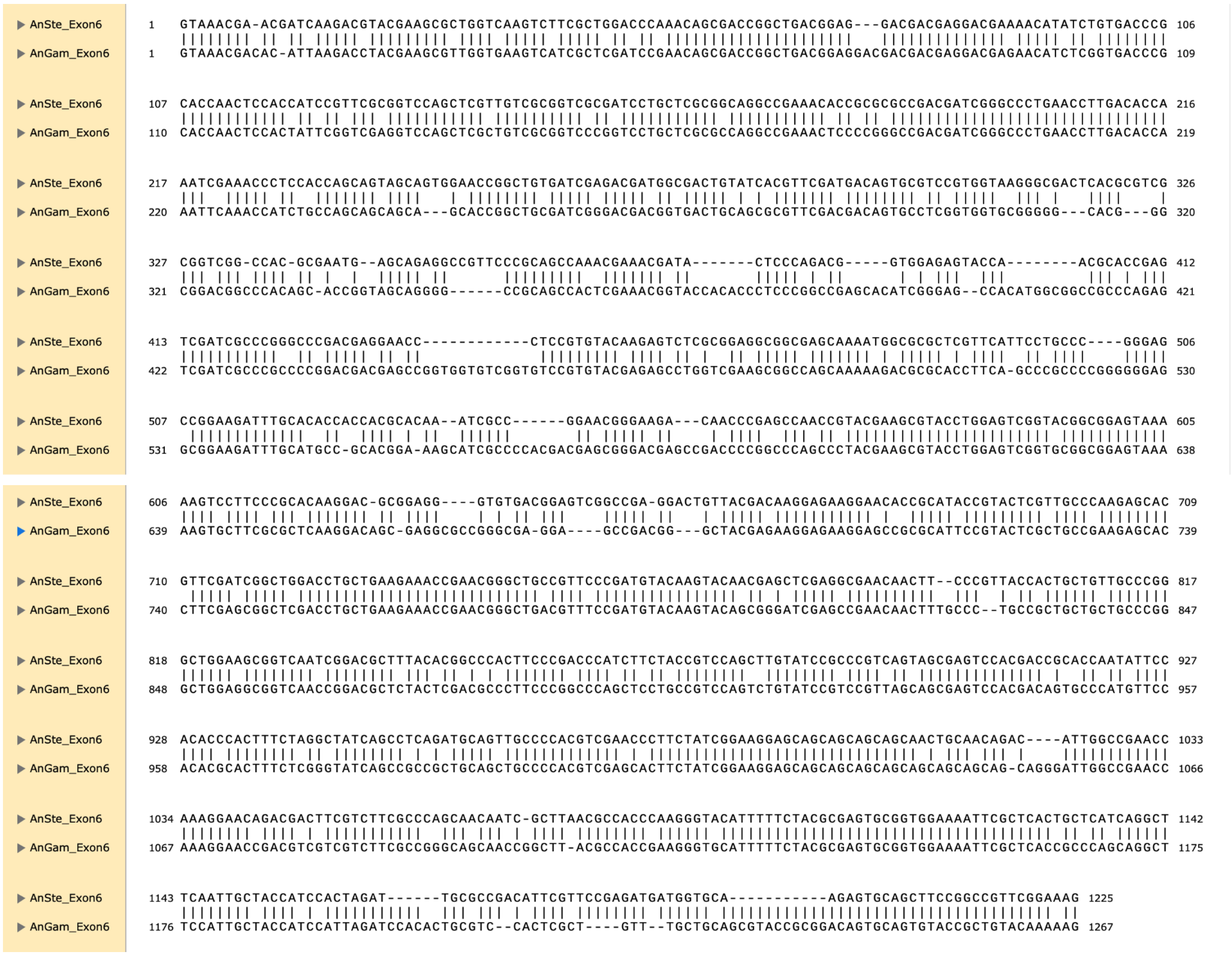

An. stephensi			
	Splice donor	Splice acceptor	Intron size
Exon 4	ACGAAG/gtaagctggcgatgat	ttatgttcaactacag/GTCAAG	575

Exon 5 within	-	acccattcggtaacag/TGTACA	-
Exon 5	CAGAAG/gtatggtaatcggcga	cgcccacgaacccaag/GTAAAC	171
Exon 6	GGAAAG/gtaagtccctgcaacc	-	-
An. gambiae			
	Splice donor	Splice acceptor	Intron size
Exon 4	ACGAAG/gtaagctggcgatgat	tttatgtttaacacag/GTCAAG	512
Exon 5 within	-	accccggcacatacag/TGTACA	-
Exon 5	CAGAAG/gtatggtaagacggcc	tgtaacccccaaaaag/GTAAAC	162
Exon 6	AAAAAG/gtaagtgtgggtagta	-	-

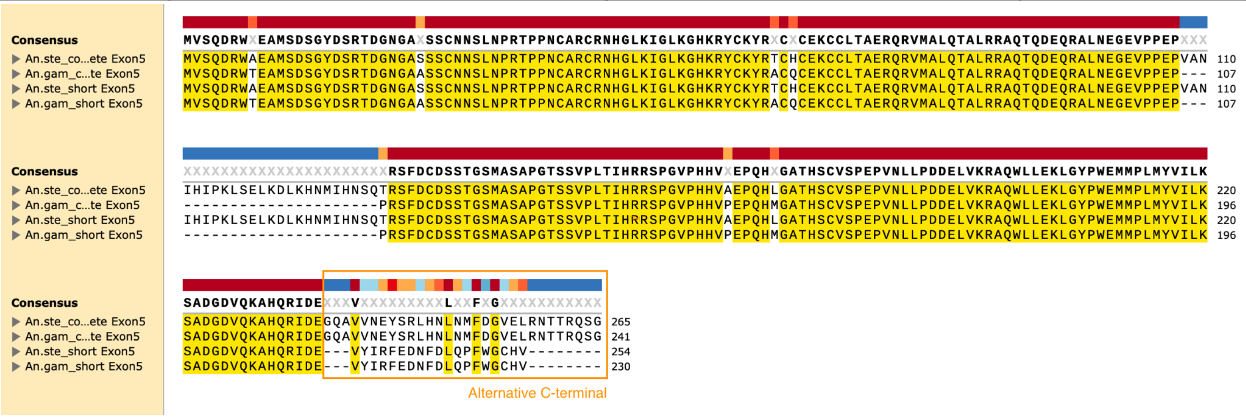

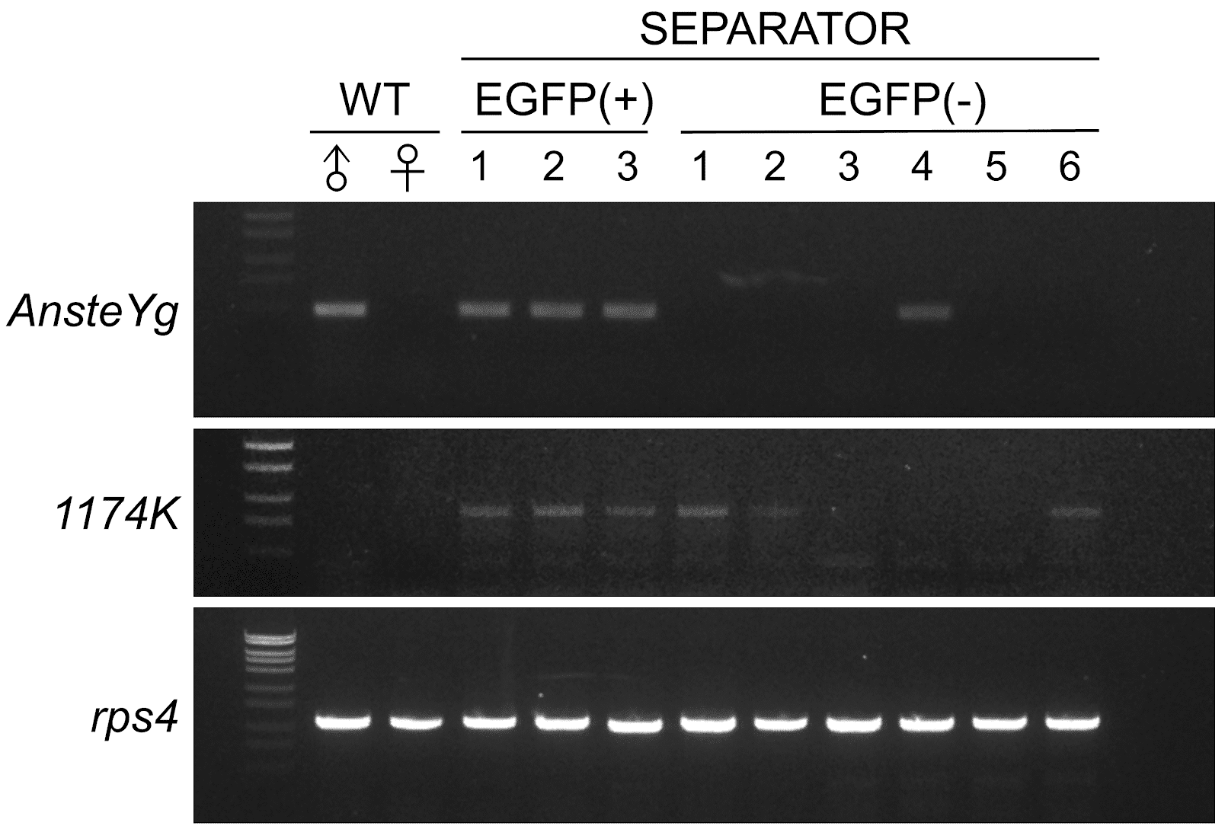

Figure S2. EGFP-positive mosquitoes are Y chromosome-containing mosquitoes.
Genomic DNA was extracted from individual mosquitoes in both the EGFP-positive and EGFP-negative groups. PCR was performed using primers specific to Y chromosome-linked genes to identify male mosquitoes among both groups.

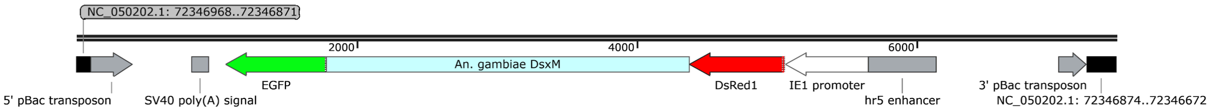

Figure S3. The full length of SEPARATOR is inserted into the *An. stephensi* genome. Confirmed PCR and sequencing results indicated that the full length of SEPARATOR is located on chromosome 2 (NC_050202.1: 72346871) in SEPARATOR mosquitoes.

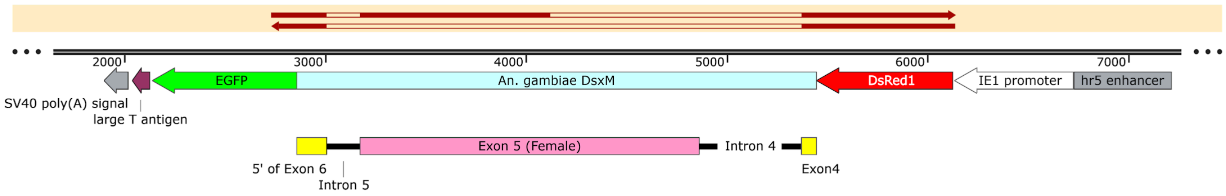

Figure S4. The sex-specific *dsx* transcripts align with sex-specific RNA splicing patterns in SEPARATOR mosquitoes.

Both male-specific and female-specific transcripts contain the DsRed coding sequence. The female-specific exon5 has been spliced out in the male-specific *dsx* transcripts and in-frame with GFP coding sequences.

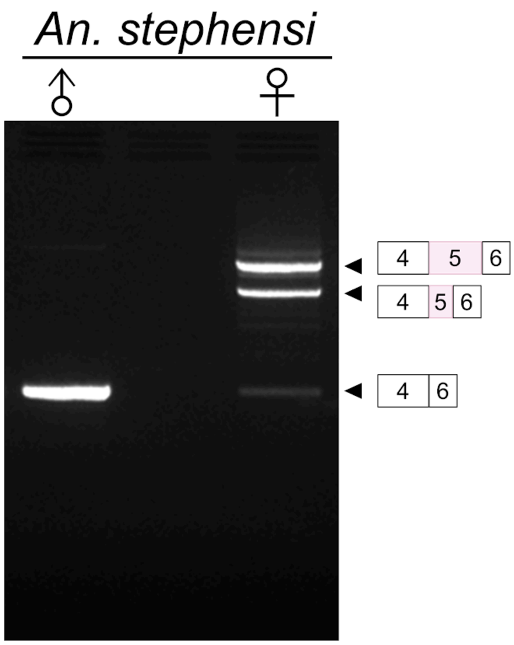

Figure S5. Two female-specific *dsx* transcripts were observed in *An. stephensi.*
Male and female wild-type *An. stephensi* mosquitoes were selected at the pupal stage, and total RNA was extracted from each group. To investigate the splicing patterns, RT-PCR was performed using specific primers targeting the exon4 and exon6 sequences. The PCR products were then subjected to agarose gel electrophoresis, followed by gel purification and sequencing to confirm the splicing patterns. The resulting splicing patterns are shown in the right panel.

Figure S6. Sequencing results related to Figure S3

```
>1174K_Male RT-PCR (1043 bp)
accaagtgacctcgagatggtgcgctcctccaagaacgtcatcaaggagttcatgcgcttcaaggtgcgcatggagggca
ccgtgaacggccacgagttcgagatcgagggcgagggcgagggccgcccctacgagggccacaacaccgtgaagctgaag
gtgaccaagggcggcccccctgcccttcgcctgggacatcctgtcccccagttccagtacggctccaaggtgtacgtgaa
gcaccccgccgacatccccgactacaagaagctgtccttccccgagggcttcaagtgggagcgcgtgatgaacttcgagg
acggcggcgtggtgaccgtgacccaggactcctccctgcaggacggctgcttcatctacaaggtgaagttcatcggcgtg
aacttcccctccgacggccccgtaatgcagaagaagaccatgggctgggaggcctccaccgagcgcctgtaccccccgcga
cggcgtgctgaagggcgagatccacaaggccctgaagctgaaggacggcggccactacctggtggagttcaagtccatct
acatggccaagaagcccgtgcagctgcccggctactactacgtggactccaagctggacatcacctcccacaacgaggac
tacaccatcgtggagcagtacgagcgcaccgagggccgccaccacctgttcctgatgatgcccctgatgtacgtcatact
aaagagcgccgatggcgatgtacaaaaagcacaccagcggatcgacgaaggtaaacgacacattaagacctacgaagcgc
tggtgaagtcatcgctcgatccgaacagcgaccggctgacggaggacgacgacgaggacgagaacatctcggtgacccgc
accaactccaccattcggtcgaggtccagctcgctgtcgatggtgagcaagggcgaggagctgttcaccggggtggtgcc
catcctggtcgagctggacggcgacgtaaacggccacaagttcagcgtgtccggcgagggcgagggcgatgccacctacg
gca

>1174K_Female  RT-PCR (1993 bp)
ttgccgtaggtggcatcgccctcgccctcgccggacacgctgaacttgtggccgtttacgtcgccgtccagctcgaccag
gatgggcaccaccccggtgaacagctcctcgcccttgctcaccatcgacagcgagctggacctcgaccgaatggtggagt
tggtgcgggtcaccgagatgttctcgtcctcgtcgtcgtcctccgtcagccggtcgctgttcggatcgagcgatgacttc
accagcgcttcgtaggtcttaatgtgtcgtttaccttctgcacagattgattgattgtagagtggagtggaaagtggtct
gctctagatcagcaccgcgcaaacggtagtgatgtagcgcaaggatttttctttttgattgatgtatgtgtgtacgcagt
attcccgcatttccccgcccccgggattgttgaaaattcccattttggctgaacaactgtgttacggaaaacaaacaacg
aacatttctactgctcttttgtactctcgatctcgatctatctctctctagccgtctctttctcttttttttgctaataac
acacacgcacgcacaattgcacaccatcggtacaggctgttggttttgattggagaaggtgacgatggcatgtgggtcgc
gcccgtaagaaaggtcgtctcagatcaccctacggtggcgcggacaaggttcatttttaataaagttgtggcacgataac
gatgacgatgttgactcaggtattggttgaacgatggttggaaggttgattggtgacgaaactagactaaactattcgct
actagtggaacacaggttgctctcctccgccttctactttgcttcattttcattttcattttcattatgcactctatta
ctccttcgcttttcagcacacgcaactgctgctgtttctcgctaaacctaacacacacacacacacttgaacgcccaatg
cacgctttcgacacttcgaagggcatccttctctcagagcaacgccggagctactacagtggagcccggactgttttgca
ggggcatgagccgagccggtgcatccctggacggggtagttccagaggggtagtgttggagtggacacactgtgtggcgg
ccattcgcgctttacaccgtcggcgcccgatctcacaagtatagctacacgtggcaaccccagaagggctgcaggtcaaa
gttgtcctcaaaccgtatgtacacttcgtcgatccgctggtgtgctttttgtacatcgccatcggcgctctttagtatga
cgtacatcaggggcatcatcaggaacaggtggtggcggccctcggtgcgctcgtactgctccacgatggtgtagtcctcg
ttgtgggaggtgatgtccagcttggagtccacgtagtagtagccgggcagctgcacgggcttcttggccatgtagatgga
cttgaactccaccaggtagtggccgccgtccttcagcttcagggccttgtggatctcgcccttcagcacgccgtcgcggg
ggtacaggcgctcggtggaggcctcccagcccatggtcttcttctgcattacggggccgtcggaggggaagttcacgccg
atgaacttcaccttgtagatgaagcagccgtcctgcagggaggagtcctgggtcacggtcaccacgccgccgtcctcgaa
gttcatcacgcgctcccacttgaagccctcggggaaggacagcttcttgtagtcggggatgtcggcggggtgcttcacgt
acaccttggagccgtactggaactgggggacaggatgtcccaggcgaagggcaggggggccgcccttggtcaccttcagc
ttcacggtgttgtggccctcgtaggggcggccctcgccctcgccctcgatctcgaactcgtggccgttcacggtgccctc
catgcgcaccttgaagcgcatgaactccttgatgacgttcttggaggagcgcaccatctcgaggtcacttggt
```

Figure S7. Sequencing results related to Figure S4

```
LOCUS       1174K gDNA confirmed PCR       7417 bp DNA     linear   UNA
27-APR-2024
DEFINITION  1174K_01.
ACCESSION   1174K_01
VERSION     .
KEYWORDS    .
SOURCE      natural DNA sequence
  ORGANISM  unspecified
REFERENCE   1  (bases 1 to 7417)
  AUTHORS   .
  TITLE     Direct Submission
  JOURNAL   Exported Sep 6, 2024 from SnapGene 7.2.0
            https://www.snapgene.com
FEATURES             Location/Qualifiers
     source          1..7417
                     /mol_type="genomic DNA"
                     /organism="unspecified"
     misc_feature    1..98
                     /label= NC_050202.1: 72346968..72346871
                     /note="Anopheles stephensi strain Indian chromosome 2,
                     UCI_ANSTEP_V1.0"
     misc_feature    99..407
                     /label=5' pBac transposon
                     /label=nonstandard type: transposon
                     /note="5' pBac"
     polyA_signal    822..943
                     /label=SV40 poly(A) signal
                     /note="SV40 polyadenylation signal"
     CDS             complement(join(1065..1778,1779..1781,1782..1784))
                     /codon_start=1
                     /product="the original enhanced GFP (Yang et al., 1996)"
                     /label=EGFP
                     /note="mammalian codon-optimized"

/translation="MVSKGEELFTGVVPILVELDGDVNGHKFSVSGEGEGDATYGKLTL

KFICTTGKLPVPWPTLVTTLTYGVQCFSRYPDHMKQHDFFKSAMPEGYVQERTIFFKDD

GNYKTRAEVKFEGDTLVNRIELKGIDFKEDGNILGHKLEYNYNSHNVYIMADKQKNGIK

VNFKIRHNIEDGSVQLADHYQQNTPIGDGPVLLPDNHYLSTQSALSKDPNEKRDHMVLL
                     EFVTAAGITLGMDELYK"
     misc_feature    1785..4372
                     /label=An. gambiae DsxM
     CDS             complement(join(4373..5044,5045..5047,5048..5050))
                     /codon_start=1
                     /product="wild-type DsRed"
```

```
                     /label=DsRed1
                     /note="mammalian codon-optimized"

/translation="MVRSSKNVIKEFMRFKVRMEGTVNGHEFEIEGEGEGRPYEGHNTV

KLKVTKGGPLPFAWDILSPQFQYGSKVYVKHPADIPDYKKLSFPEGFKWERVMNFEDGG

VVTVTQDSSLQDGCFIYKVKFIGVNFPSDGPVMQKKTMGWEASTERLYPRDGVLKGEIH

KALKLKDGGHYLVEFKSIYMAKKPVQLPGYYYVDSKLDITSHNEDYTIVEQYERTEGRH
                     HLFL"
     promoter        complement(5061..5652)
                     /label=IE1 promoter
                     /note="promoter of the ie1 gene from the baculovirus
                     Autographa californica"
     enhancer        5656..6138
                     /gene="Autographa californica hr5"
                     /label=hr5 enhancer
                     /note="baculovirus early transcription enhancer"
     misc_feature    7014..7214
                     /label=3' pBac transposon
                     /label=nonstandard type: transposon
                     /note="3' pBac"
     misc_feature    7215..7417
                     /label=NC_050202.1: 72346874..72346672
                     /note="Anopheles stephensi strain Indian chromosome 2,
                     UCI_ANSTEP_V1.0"
ORIGIN
        1 ccctacccat cttgatgtga taatagggac ttcgtaaaga aatttaagaa ctgaaagaga
       61 agaaatgtta caccattagt gtcggtgggc ttgtttaacc ctagaaagat agtctgcgta
      121 aaattgacgc atgcattctt gaaatattgc tctctctttc taaatagcgc gaatccgtcg
      181 ctgtgcattt aggacatctc agtcgccgct tggagctccc gtgaggcgtg cttgtcaatg
      241 cggtaagtgt cactgatttt gaactataac gaccgcgtga gtcaaaatga cgcatgatta
      301 tcttttacgt gacttttaag atttaactca tacgataatt atattgttat ttcatgttct
      361 acttacgtga taacttatta tatatatatt ttcttgttat agatatcgtg actaatatat
      421 aataaaatgg gtagttcttt agacgatgag catatcctct ctgctcttct gcaaagcgat
      481 gacgagcttg ttggtgagga ttctgacagt gaaatatcag atcacgtaag tgaagatgac
      541 gtccagagcg atacagaaga agcgtttata gatgaggtac atgaagtgca gccaacgtca
      601 agcggtagtg aaatattaga cgaacaaaat gttattgaac aaccaggttc ttcattggct
      661 tctaacagaa tcttgacctt gccacagagg actattagag gtaagaataa acattgttgg
      721 tcaacttcaa agtccacgag gcgtagccga gtctctgcac tgaacattgt cagatccgag
      781 atcggccggc ctaggcgcgc cgtacgcgta tcgataagct ttaagataca ttgatgagtt
      841 tggacaaacc acaactagaa tgcagtgaaa aaaatgcttt atttgtgaaa tttgtgatgc
      901 tattgcttta tttgtaacca ttataagctg caataaacaa gttaacaaca acaattgcat
      961 tcattttatg tttcaggttc agggggaggt gtgggaggtt ttttaaagca agtaaaacct
     1021 ctacaaatgt ggtatggctg attatgatct agagtcgcgg ccgcttactt gtacagctcg
     1081 tccatgccga gagtgatccc ggcggcggtc acgaactcca gcaggaccat gtgatcgcgc
     1141 ttctcgttgg ggtctttgct cagggcggac tgggtgctca ggtagtggtt gtcgggcagc
     1201 agcacggggc cgtcgccgat gggggtgttc tgctggtagt ggtcggcgag ctgcacgctg
```

1261 ccgtcctcga tgttgtggcg gatcttgaag ttcaccttga tgccgttctt ctgcttgtcg
1321 gccatgatat agacgttgtg gctgttgtag ttgtactcca gcttgtgccc caggatgttg
1381 ccgtcctcct tgaagtcgat gcccttcagc tcgatgcggt tcaccagggt gtcgccctcg
1441 aacttcacct cggcgcgggt cttgtagttg ccgtcgtcct tgaagaagat ggtgcgctcc
1501 tggacgtagc cttcgggcat ggcggacttg aagaagtcgt gctgcttcat gtggtcgggg
1561 tagcggctga agcactgcac gccgtaggtc agggtggtca cgagggtggg ccagggcacg
1621 ggcagcttgc cggtggtgca gatgaacttc agggtcagct tgccgtaggt ggcatcgccc
1681 tcgccctcgc cggacacgct gaacttgtgg ccgtttacgt cgccgtccag ctcgaccagg
1741 atgggcacca ccccggtgaa cagctcctcg cccttgctca ccatcgacag cgagctggac
1801 ctcgaccgaa tggtggagtt ggtgcgggtc accgagatgt tctcgtcctc gtcgtcgtcc
1861 tccgtcagcc ggtcgctgtt cggatcgagc gatgacttca ccagcgcttc gtaggtctta
1921 atgtgtcgtt taccttttttg ggggttacaa ggggcggtgc aaaaagagag agagagagag
1981 aaaggggggt gggataaaaa gaaagcggaa aatcgattag caaagttcgc attcagccct
2041 cgtcggacgg cgttatggtg gcgaccgtga ctcgctcaaa ggccgtctta ccataccttc
2101 tgcacagatt gattgattgt agagtggagt ggaaagtggt ctgctctaga tcagcaccgc
2161 gcaaacggta gtgatgtagc gcaaggattt ttctttttga ttgatgtatg tgtgtacgca
2221 gtattcccgc atttccccgc ccccgggatt gttgaaaatt cccattttgg ctgaacaact
2281 gtgttacgga aaacaaacaa cgaacatttc tactgctctt ttgtactctc gatctcgatc
2341 tatctctctc tagccgtctc tttctctttt tttgctaata acacacacgc acgcacaatt
2401 gcacaccatc ggtacaggct gttggttttg attggagaag gtgacgatgg catgtgggtc
2461 gcgcccgtaa gaaaggtcgt ctcagatcac cctacggtgg cgcggacaag gttcattttt
2521 aataaagttg tggcacgata acgatgacga tgttgactca ggtattggtt gaacgatggt
2581 tggaaggttg attggtgacg aaactagact aaactattcg ctactagtgg aacacaggtt
2641 gctctcctcc gccttctact tttgcttcat tttcattttc attttcatta tgcactctat
2701 tactccttcg cttttcagca cacgcaactg ctgctgtttc tcgctaaacc taacacacac
2761 acacacactt gaacgcccaa tgcacgcttt cgacacttcg aagggcatcc ttctctcaga
2821 gcaacgccgg agctactaca gtggagcccg gactgttttg caggggcatg agccgagccg
2881 gtgcatccct ggacggggta gttccagagg ggtagtgttg gagtggacac actgtgtggc
2941 ggccattcgc gctttacacc gtcggcgccc gatctcacaa gtatagctac acgtggcaac
3001 cccagaaggg ctgcaggtca aagttgtcct caaaccgtat gtacactgta tgtgccgggg
3061 ttttcggggt tttcgacgca attacgtacc cgccggggcg tgggccacat agaaaagaac
3121 gcaaagaaca agaaatatac agatgataca aaaggggtgt gattcattgt gtcgattggt
3181 tttttgttgt tgtttgttgg tttggttgca gagattgcat gggagaaagc gggcttgtgg
3241 cagtacgaat acccacgatc ggcggaacgc tacgcaagta cgcgcaaagt cgtgcgtcca
3301 gagcgttcga ggagcagcga ccacctgctc taggatctac tatgtacaaa catgctgcag
3361 tgttttctgt gtgtgtatag gccgtttctt gaacgggacg accactgttt tgtttttgct
3421 gctccgattt aacactagaa tttccccgcc ctctccccga gatgccggac gcaccgcacc
3481 agtgcagcta ggctgccgac ccattctgct gcccttctgt accgtggtgc ggctctctcg
3541 ctccactcct taaacactag tttgaactta tcggcatcag ttgcgcacgc ggcttgattt
3601 aaaatagcac agaactattg aattcgtttc accaaacaca catacacaca cccacataca
3661 aagatacgga cagttacagt ggtgcggaaa gtttatcatc cactctgacg ggtggtattg
3721 cgcaactcca cgccatcaaa catgttcaga ttatgcaatc gtgagtattc gttgaccacc
3781 gcttgacctg tgttaaacat aaatgaatgg aaaggtaagg ctttgaaggt cactgctgct
3841 ggctgacgga attcacaatt ttgtttttga ttttgttttt tttatatatc gaattttgaa
3901 gtcagtgaac gtggcataac accatatgcc gctaccttca agatgcagat actcctaact
3961 tctcgtgtct gagctagcta acttaacatg ttggattgaa cgacatttca atgagaacag
4021 tatacataaa ctaaccctag acgattaaaa cattcttggg agttgagacg acaatgttaa
4081 acactaatac gcaggtaact taagatcatc ttatcctagg aaaaattgcc ttcaactgaa
4141 acctttcctt aacagctgca tcatgaggta gagtctctcg caaaaaagaa cacgtttttg

4201 ctacaccctg tgaccactgg acccggtgct aggcacagta gatgtctgac acggtgatga
4261 aagtgatgtc gaacgacacc atcatcgcca gcttaccttc gtcgatccgc tggtgtgctt
4321 tttgtacatc gccatcggcg ctctttagta tgacgtacat caggggcatc atcaggaaca
4381 ggtggtggcg gccctcggtg cgctcgtact gctccacgat ggtgtagtcc tcgttgtggg
4441 aggtgatgtc cagcttggag tccacgtagt agtagccggg cagctgcacg ggcttcttgg
4501 ccatgtagat ggacttgaac tccaccaggt agtggccgcc gtccttcagc ttcagggcct
4561 tgtggatctc gcccttcagc acgccgtcgc gggggtacag gcgctcggtg gaggcctccc
4621 agcccatggt cttcttctgc attacggggc cgtcggaggg gaagttcacg ccgatgaact
4681 tcaccttgta gatgaagcag ccgtcctgca gggaggagtc ctgggtcacg gtcaccacgc
4741 cgccgtcctc gaagttcatc acgcgctccc acttgaagcc ctcggggaag gacagcttct
4801 tgtagtcggg gatgtcggcg gggtgcttca cgtacacctt ggagccgtac tggaactggg
4861 gggacaggat gtcccaggcg aagggcaggg ggccgccctt ggtcaccttc agcttcacgg
4921 tgttgtggcc ctcgtagggg cggccctcgc cctcgccctc gatctcgaac tcgtggccgt
4981 tcacggtgcc ctccatgcgc accttgaagc gcatgaactc cttgatgacg ttcttggagg
5041 agcgcaccat ctcgaggtca cttggttgtt cacgatcttg tcgccgccag tgtcaacttg
5101 caactgaaac aatatccaac atgaacgtca atttatactg ccctaatggc gaacacgata
5161 acaatatttc ttttattatg ccctctaaaa ccaacgcggt tatcgtttat ttattcaaat
5221 tagatataga acatccgccg acatacaatg ttaatgcaaa aacgcgtttg gtgagcggat
5281 acgaaaacag tcggccgata aacattaatc tgaggtcgat aacaccgtcc ttgaacggaa
5341 cacgaggagc gtacgtgatc agctgcattc gcgcgccgcg cctttatcga gatttatttg
5401 catacaacaa gtacactgcg ccgttgggat ttgtggtaac gcgcacacat gcagagctgc
5461 aagtgtggca cattttgtct gtgcgcaaaa cctttgaagc caaaagtacg aggtccgtta
5521 cgggcatgct agcgcacacg gacaatggac ccgacaaatt ctacgccaag gatttaatga
5581 taatgtcggg caacgtatcc gttcatttta tcaataacct acaaaaatgt cgcgcgcatc
5641 acaaagacat cgacgcgcgt agaattctac ccgtaaagcg agtttagtta tgagccatgt
5701 gcaaaacatg acatcagctt ttatttttat aacaaatgac atcatttctt gattgtgttt
5761 tacacgtaga attctactcg taaagcgagt tcagttttga aaaacaaatg acatcatctt
5821 tttgattgtg ctttacaagt agaattctac ccgtaaatca agttcggttt tgaaaaacaa
5881 atgagtcata ttgtatgata tcatattgca aaacaaatga ctcatcaatc gatcgtgcgt
5941 tacacgtaga attctactcg taaagcgagt ttatgagccg tgtgcaaaac atgacatcat
6001 ctcgatttga aaaacaaatg acatcatcca ctgatcgtgc gttacaagta gaattctact
6061 cgtaaagcca gttcggttat gagccgtgtg caaaacatga catcagctta tgactcatac
6121 ttgattgtgt tttacgcggt ttaaaccccg ggcgagctcg aattaaccat tgtgggaacc
6181 gtgcgatcaa acaaacgcga gataccggaa gtactgaaaa acagtcgctc caggccagtg
6241 ggaacatcga tgttttgttt tgacggaccc cttactctcg tctcatataa accgaagcca
6301 gctaagatgg tatacttatt atcatcttgt gatgaggatg cttctatcaa cgaaagtacc
6361 ggtaaaccgc aaatggttat gtattataat caaactaaag gcggagtgga cacgctagac
6421 caaatgtgtt ctgtgatgac ctgcagtagg aagacgaata ggtggcctat ggcattattg
6481 tacggaatga taaacattgc ctgcataaat tcttttatta tatacagcca taatgtcagt
6541 agcaagggag aaaaggttca aagtcgcaaa aaatttatga gaaaccttta catgagcctg
6601 acgtcatcgt ttatgcgtaa gcgtttagaa gctcctactt tgaagagata tttgcgcgat
6661 aatatctcta atattttgcc aaatgaagtg cctggtacat cagatgacag tactgaagag
6721 ccagtaatga aaaaacgtac ttactgtact tactgcccct ctaaaataag gcgaaaggca
6781 aatgcatcgt gcaaaaaatg caaaaaagtt atttgtcgag agcataatat tgatatgtgc
6841 caaagttgtt tctgactgac taataagtat aatttgtttc tattatgtat aagttaagct
6901 aattacttat tttataatac aacatgactg tttttaaagt acaaaataag tttatttttg
6961 taaaagagag aatgtttaaa agttttgtta ctttatagaa gaaattttga gtttttgttt
7021 tttttttaata aataaataaa cataaataaa ttgtttgttg aatttattat tagtatgtaa
7081 gtgtaaatat aataaaactt aatatctatt caaattaata aataaacctc gatatacaga

```
7141 ccgataaaac acatgcgtca attttacgca tgattatctt taacgtacgt cacaatatga
7201 ttatctttct agggttaaca cgtggaagtg agtgtgaact ttgggaacgt ttcgagaccg
7261 tatcgaaagg cgtgtggaat ttatattcga cgttaagcga atacattttc ccaaaggcca
7321 gtagacgggg cacactaaat ggaaaactaa atacgaaaaa tgggcaaaaa ggtagaaaat
7381 ctgagcctta ccaaagcaca agctatgaac agtttag
//
```
