# Supplementary material for: Establishing a dominant early larval sex-selection strain in the Asian malaria vector Anopheles stephensi
Source: Infect Dis Poverty. 2024 Nov 11;13:83. doi: 10.1186/s40249-024-01256-7 (PMC11552218; doi:10.1186/s40249-024-01256-7)
Supplement: Supplementary file 2 — Supplementary Material 2: Table S1. The sex sorting of SEPARATOR mosquitoes. Table S2. Sequences of primers used in this study [file 40249_2024_1256_MOESM2_ESM.pdf]

Table S1. The sex sorting of SEPARATOR mosquitoes during the initial 15 generations

		GFP+			GFP-		
**Number of generation**	**Number of counted mosquitoes**	**Number of male**	**Number of female**	**Male proportion**	**Number of male**	**Number of female**	**Female proportion**
G0	19	3	0	100%	0	16	100%
G1	19	12	0	100%	0	7	100%
G2				NA			
G3				NA			
G4				NA			
G5	134	43	0	100%	24	67	74%
G6	89	39	0	100%	8	42	84%
G7	105	48	0	100%	5	52	91%
G8	97	50	0	100%	3	44	94%
G9	107	50	0	100%	1	56	98%
G10	71	38	0	100%	0	33	100%
G11	95	49	0	100%	0	46	100%
G12	95	49	0	100%	0	46	100%
G13	280	143	0	100%	0	137	100%
G14	877	451	0	100%	0	426	100%
G15	1150	581	0	100%	0	569	100%

*NA means not analyzed

Table S2. Sequences of primers and gBlock fragment used in this study

Name	Sequence	Amplicon	Usage	Note
1174K AgDsx E4 F	gagggccgccaccacctgttcctgATGATGCCCCTGATGTACGTCATACTAAAGAG	Hr5Ie1 to 5' of Intron4	Dsx splicing module assembly	
1174K AgDsx E6-GFP R	tcctcgcccttgctcaccatCGACAGCGAGCTGGACCTCGACCGAATAG			
1174I ie1-F2	ggcggcgacaagatcgtgaacaaccaagtgac	Hr5ie1 to EGFP	RT-PCR of the SEPERATOR splicing module	figure 1.D
1174I GFP-R2	cagatgaacttcagggtcagcttgccgtagg			
AS_YG2_F	TGCCGGACATGACATTTG	GenBank: KC822953	Y chromosome marker	figure S2
AS_YG2_R	TCAATGCGAACAGAAGGCTAA			
1179C.10	gtttaggtcgagtaaagtttCCCTAGAAAGATAGTCTGC	sv40 to pBAC arm	The marker of SEPERATOR	figure S2
934.S26	AAAGCAATAGCATCACAAATTTCA			
AS_RPS4-F	GAGTCCATCAAAGGAGAAAGTCTAC	RPS4	Genomic reference	figure S2
AS_RPS4-R	TAGCTGGCGCATCAGGTAC	RPS4		
1179_dsx_c1	GCATCTCGGAGCTACTCATTC	*An. stephensi dsx* Exon2/3 junction to Exon6	RT-PCR of the *dsx* splicing	figure S5
1179_dsx_c2	CTGGTGGAGGGTTTCGATTT			
pBAC_L1	TGGCTCTTCAGTACTGTCAT	pBAC left arm	Identify the SEPARATOR insertion site in the genome	
pBAC_L2	TACGCATGATTATCTTTAACGTA			
pBAC_L3	GCATGTGTTTTATCGGTC	pBAC left arm with pBAC_L1	The same as previous	
pBAC_L1nest	CACTTCATTTGGCAAAATAT	within amplicon of pBAC_L1/L2	The same as previous	
pBAC_L2nest	GTCACAATATGATTATCTTTCTAGG			
pBAC_R1	GACACTTACCGCATTGACAAG	pBAC rightarm	The same as previous	
pBAC_R2	CAAAGTCCACGAGGCGTAG			
pBAC_R1nest	ACTGAGATGTCCTAAATGCACAG	within amplicon of pBAC_R1/R2	The same as previous	
pBAC_R2nest	CCGAGTCTCTGCACTGAAC			
1174K Piggybac F	cgtccatttcgtccgcagtc	Full length of SEPARATOR in SEPARATOR mosquitoes	Verifying that SEPARATOR is located on chromosome 2	figure S3
1174K Piggybac R	tggatttccaggttgctttg			
